# The Digital Psychiatrist: In Search of Evidence-Based Apps for Anxiety and Depression

**DOI:** 10.3389/fpsyt.2019.00831

**Published:** 2019-11-15

**Authors:** Jamie M. Marshall, Debra A. Dunstan, Warren Bartik

**Affiliations:** School of Psychology, Faculty of Medicine and Health, University of New England, Armidale, NSW, Australia

**Keywords:** mHealth, mental health, apps, app store, depression, anxiety, e-mental health, digital

## Abstract

One of the biggest growth areas in e-mental health resources has been the development and use of mobile mental health apps for smartphones and tablet devices. Such apps are being downloaded at increasing rates, but there have been questions about their efficacy and the research methodologies used to examine this. A review of the major app marketplaces, the Apple App Store and Google Play store, was conducted to locate apps claiming to offer a therapeutic treatment for depression and/or anxiety, and have research evidence for their effectiveness, according to their app store descriptions. App store descriptions were also analyzed to determine whether the app had been developed with mental health expert input; whether they had been developed in association with a government body, academic institution, or medical facility; and, whether or not they were free to download. Overall, 3.41% of apps had research to justify their claims of effectiveness, with the majority of that research undertaken by those involved in the development of the app. Other results indicated that 30.38% of shortlisted apps claimed to have expert development input; 20.48% had an affiliation with a government body, academic institution, or medical facility; and, 74.06% were free to download. Future research must consider other methodologies that may facilitate more research being completed on a greater number of apps, and future development needs to incorporate greater levels of input by mental health experts. Ways in which app stores could play a key role in encouraging more scientific research into the effectiveness of the mental health apps they sell are discussed.

## Introduction

### Smartphones and Apps

Smartphones are mobile/cellular phones capable of connecting to the Internet and performing similar functions to computers. Smartphone applications (apps) are software programs designed to perform specific tasks on smartphones. Through the use of apps, smartphones can perform many functions, including monitoring, assessing, and treating physical and mental health conditions. On a global scale, there are over 5.2 billion people who own a smartphone (1).

The proliferation of smartphone apps has led to digital solutions for a myriad of situations. The largest app marketplaces are the Apple App store and Google Play store. Developers pay to have their apps available for download from these stores, which receive commissions from downloaded apps. Apps appear in searches based on search terms, but information about the complex algorithms used to determine what apps appear higher in a search than others is not publicly known. In 2018, global expenditure on downloaded apps was approximately $US92.1 billion (2), highlighting the lucrative business of being an app marketplace.

### Health and Mental Health Apps

Health apps (including those related to mental health) are one of the fastest growing categories of apps (3). Over two-thirds of American adults are willing to use their smartphone to help manage their health (4), and about 60% of people who own a smartphone have downloaded at least one health app (5). There are approximately 318,000 health apps currently available (6). Of these, more than 10,000 relate to mental health (7).

Research has shown that people with mental illness increasingly own smartphones and other mobile devices, and are interested in using these to monitor their mental health (5, 8, 9). However, there is a large gap between interest in and use of such apps (9, 10), and variability in knowledge about the capabilities of existing mental health apps (11). Further, people living with a mental illness can have distinctly negative attitudes towards an app’s ability to manage sensitive information associated with mental health (12). These findings indicate that consumers’ attitudes and responses to mental health apps are diverse.

Currently, the main ways individuals choose mental health apps are *via* ratings and reviews in the app stores (13), or through comments made through social media or word of mouth (14). However, price is also important, showing a negative correlation with downloads, and that lower priced mental health apps have consistently higher ratings than higher priced apps (13). Over half of those who have downloaded a health app value ease of use over trustworthiness of the app (15), suggesting that demonstrated effectiveness is not an important consideration for many consumers.

### Potential Benefits of Mental Health Apps

There are many potential benefits of using mental health apps for alleviating depression and anxiety. These include portability, immediacy and accessibility. These features may be of particular benefit to rural populations; people on waiting lists for face-to-face services; or, difficult-to-engage groups such as teenagers (16). Other advantages include affordability, convenience, and anonymity. These benefits may have specific relevance to lower socioeconomic groups who find traditional treatment cost-prohibitive (17), or those who fear stigmatization. Mobile apps also offer convenient ways of practicing strategies learned in face-to-face therapy, and may incorporate reminders that can be set to increase treatment and medication compliance. By offering effective options to those with milder psychiatric symptoms, the burden on traditional mental health services could be reduced (18). These potential benefits provide compelling reasons to pursue research on the efficacy of mental health apps.

### The Current Study

The focus of this app store search was on apps that claimed to address depression and/or anxiety symptoms. These conditions are the most common forms of mental illness (19), and many of their symptoms and causes overlap. While the potential benefits of apps for depression and anxiety are many, there are also possible disadvantages and dangers in using untested and potentially ill-informed apps that may risk doing harm to an individual (e.g. suggesting an individual undertake an activity that is not evidence-based that may have adverse consequences such as drinking alcohol to relax, or taking long-term higher doses of addictive medication to improve sleep). Presently, there is no reliable approximation of the proportion of apps for depression or anxiety that are available in the app stores which have scientific evidence of efficacy, or that have been developed with input from mental health professionals or in collaboration with a government body, academic institution, or medical facility. Further, given that individuals rate free apps more highly than those that incur a cost, and that one of the potential benefits of mental health apps is that people from lower socioeconomic backgrounds may have access to mental health treatment they might not otherwise be able to afford, it is important to know what proportion of mental health apps are available free of charge to the consumer. To address these questions, this mini-review examined apps that claim to offer a therapeutic intervention for reducing symptoms of depression or anxiety and are listed in the Apple App Store and Google Play store under one of the search terms used. The research questions were: What proportion of apps for depression and/or anxiety

1. … claim to have research evidence for their effectiveness?
2. … have involved a mental health expert in their development?
3. … have been developed in affiliation with an academic institution, medical facility, or other government-funded body?
4. … are free to download?

## Methods

The methodology for this mini-review was informed by the AMSTAR (20) and PRISMA (21) protocols for systematic reviews. The coding regime used was based on Alyami et al. (22) and Shen et al. (23), both of whom conducted app marketplace searches for mental health apps.

Four researchers, including the lead author, searched the Apple App Store and Google Play store, in December 2018. Previous research had shown that these two marketplaces account for over 90% of available apps for depression (23).

A total of 19 key word searches were used for each marketplace across all categories: mental health, depression, anxiety, wellbeing, happiness, psychological distress, positive psychology, suicide, mental illness, CBT, cognitive behavior therapy, cognitive behavior therapy, ACT, acceptance and commitment therapy, DBT, dialectical behavior therapy, dialectical behavior therapy, IPT, and interpersonal therapy.

Apps were shortlisted and data were extracted based on their descriptions in the app stores (that is, apps were not downloaded individually). Data relating to organization affiliation and the involvement of mental health professionals was coded as shown in **Table 1**. Information about the cost of the app and whether the app claimed any research on effectiveness were also noted from descriptions in the app store. Researchers then located all the claimed research, verifying its existence, through searches made in Google Scholar. To be consistent with how a consumer would go about finding an app, the supporting literature was not identified first.

**Table 1:** Coding used for app store search.

Variable	Code	Description
Organizational affiliation	UNI	University. Produced in association with a university or other academic institution.
	MEDC	Medical Center. Produced in association with a medical institution or hospital.
	GOVT	Government. Produced in affiliation with a government institution.
	INST	Institution. An explicit association (i.e. foundation, center, non-government organization, etc.).
	OTHER	Other. There is a clear but unclassifiable affiliation (e.g. a company), but not a “.com”.
	INSUFF	Insufficient. The affiliation cannot be confirmed by available information.
Content source	EXP	Expert. Developed by/with an accredited medical or allied health professional, or a recognized institution.
	EXT	External source. From a specific external source (e.g. DSM, recognized inventory, association etc.), but not based on or inspired by a theory/practice (e.g. CBT).
	LAY	Layperson. Source identified but no credential mentioned. Non-medical expertise clearly indicated by detailed bio or qualifier (e.g. years of experience).
	PLE	Person lived experience. Indication that the app is developed by a person with lived experience.
	INSUFF	Insufficient. No direct information provided about origin of information.

Inclusion criteria were:

1. The app language is English;
2. Reported research is in English and published in a peer-reviewed journal;
3. Supporting research is an intervention study with outcomes measuring depression and/or anxiety symptoms;
4. The app offers a therapeutic treatment, not just symptom monitoring, thought recording, or diagnostic tools (although apps could have any of these as part of a therapeutic treatment). A therapeutic treatment was viewed as one that takes a comprehensive and broad approach to treating anxiety and/or depression;
5. The research did not examine the app as an adjunct to other types of therapy, such as receiving therapist support.

An example of statements from a description in the app store that led the app to be identified as one that offered a therapeutic treatment for anxiety and/or depression (for Destressify):

“… skills for dealing with thoughts, emotions, beliefs that induce stress or anxiety …”

“… this comprehensive program …”

“… the core plan consists of 14 key practices …”

“… meditations or mindfulness exercises … schedule these practices and get reminders …”

“… latest neuroscientific research … neural pathways of the brain rewire themselves …”

An example of statements from a description in the app store that led the app to not be included as one that offered a therapeutic treatment for anxiety and/or depression (for Anxiety Panic Attacks Game—the fifth highest ranked app after typing in “anxiety” to the Apple App Store’s search box):

“Play now the #1 addictive game! Be careful not to pass through the obstacle, or you’ll have to start again. When you need relaxation, diversion or just a moment of distraction enjoy with your hero. Pass through different obstacles, collect stars and buy new characters!”

Apps that were available in both app stores were only counted once.

## Results

The app marketplace search resulted in 293 apps shortlisted for closer inspection based on their app store description of offering a therapeutic treatment for reducing depression and/or anxiety symptoms.

Examination of each shortlisted app’s description against the inclusion criteria identified 10 apps with evidence of a research base for efficacy (see **Table 2**), representing 3.41% of the total number of shortlisted apps across both app marketplaces. When analyzing each app store separately, differences between the two were negligible: The Apple App Store had 3.05% of its depression and anxiety apps having published research evidence and Google Play store had 4.17%. Of the 10 apps that had research support, in only three cases (1.02% of the shortlisted apps) was the research independent (i.e. conducted by an institution or individuals that were not involved in the app’s development and/or who would not benefit financially from the app).

**Table 2:** Research evidence for the effectiveness of apps that offer a therapeutic treatment for anxiety and/or depression.

Article	App name	Design	Participants	Outcome	Time/Length	Statistical analysis
Lee and Jung (24) *	Destressify	RCT	77 participants in intervention group, 86 in waitlist control group; mean age 20.6 years	Stress, anxiety, and depression	Intervention period 4 weeks; no long-term follow-up	ANCOVA/MANCOVA; detailed in article
Christoforou et al. (25)	Agoraphobia Free	RCT	73 participants in intervention group, 69 in other app “control” group (no waitlist group), mean age 39.7 years	Anxiety (agoraphobia)	Intervention period 12 weeks; no long-term follow-up	Linear mixed model; detailed in article
Kinderman et al. (26)	Catch It	Feasibility/pilot study	285 participants (no control group); mean age 48.0 years	Positive/negative mood	Intervention period 6 weeks maximum, but varied amongst participants; no long-term follow-up	ANOVA; detailed in article
Carey et al. (27)	Mindsurf	Feasibility/pilot study	23 participants (no control group); no mean age given	Anxiety, depression	Intervention period 2 weeks; no long-term follow-up	ANOVA; inadequate detail in article
Kuhn et al. (28)	PTSD Coach	RCT	62 participants in intervention group, 58 in waitlist control group; mean age 39.0 years	Anxiety (posttraumatic stress disorder), depression	Intervention period 3 months; 3 months long-term follow-up	Repeated measures ANOVAs; detailed in article
Bakker et al. (29)	MoodMission	RCT	56 participants in intervention group 1, 56 in intervention group 2, 50 in intervention group 3, 64 in waitlist control group; mean age 34.0 years	Anxiety, depression	Intervention period 30 days; no long-term follow-up	ANOVA; detailed in article
Roepke et al. (30)	SuperBetter	RCT	93 participants in intervention group 1, 97 in intervention group 2, 93 in waitlist control group; mean age 40.2 years	Anxiety, depression, life satisfaction	Intervention period 4 weeks; 6 weeks long-term follow-up	Hierarchical Linear Modeling; detailed in article
Stiles-Shields et al. (31)	Thought Challenger	RCT	10 participants in intervention group 1, 10 in intervention group 2, 10 in waitlist control group; no mean age given	Depression	Intervention period 6 weeks; 4 weeks long-term follow-up	Repeated measures ANOVA; detailed in article
Flett et al. (32) *	Smiling Mind	RCT	58 participants in intervention group, 67 in placebo control group; mean age 20.1 years	Stress, anxiety, depression, flourishing	Intervention period 40 days; no long-term follow-up	Multiple regression; detailed in article
Flett et al. (32) *	Headspace	RCT	67 participants in intervention group, 67 in placebo control group (same control group as above study); mean age 20.1 years	Stress, anxiety, depression, flourishing	Intervention period 40 days; no long-term follow-up	Multiple regression; detailed in article

*Independent research. RCT, Randomized controlled trial; ANOVA, Analysis of variance; ANCOVA, Analysis of covariance; MANCOVA, Multivariate analysis of co-variance.

The published research articles varied in design and methodology. Seven were randomized controlled trials and two were feasibility/pilot studies without control groups. A total of 1017 participants were in intervention groups, and 447 were in control groups. The mean age across all participants was 32.7 years. Intervention periods varied between 2 and 12 weeks, with the longest period of follow-up being 3 months.

In contrast, 30.38% (89/293) of apps claimed to have developmental input from a mental health expert and 20.48% (60/293) claimed to be affiliated with an organization, institution, government body, medical center, or university.

Finally, it was found that 74.06% (217/293) of apps offering a therapeutic treatment for anxiety and/or depression were free to download.

## Discussion

A search of the Apple App Store and Google Play store revealed a total of 293 apps claiming to offer a therapeutic treatment for depression and/or anxiety. Of those 293 apps, only 3.41% had published research on their effectiveness. One other app store review has previously examined what proportion of mental health apps had published evidence, but focused only on social anxiety apps, and failed to find any such apps with published evidence (22). This is the first app store review to examine the proportion of depression and anxiety apps in general that have published research corroborating claims of efficacy.

This mini-review found that the small amount of research to date has been completed mainly by individuals and organizations who have an affiliation with the app; either through its development, or being on its staff or board, or otherwise being in a position to financially benefit when the app is sold. Only 1.02% of the 293 apps had support shown in independent research. With so little independent research supporting favorable evaluations, questions of researcher bias and conflicts of interest inevitably arise.

The nine published research articles (examining the 10 apps that were first found in the app stores) are of varying quality. Most do not have long-term follow-up data, with one study having a 3-month follow-up, and two further studies having 4- and 6-week follow-up data. This reveals that even in this small sample of research, taken from a large number of apps found in the first instance, there is very little evidence to date that apps for anxiety and depression can have positive long-term effects on their users. Furthermore, the intervention periods vary enormously between studies, providing little in the way of guidance about optimal dosage/usage. This issue is reinforced by the non-existence of any replication studies that might manipulate dosage/usage.

Less than one third (30.38%) of apps claimed to have development input from a mental health expert, meaning that over two-thirds of apps for treating depression and/or anxiety were developed without any professional input. Previous research has produced similar findings of professional input of 38.3% (23) and 34.21% (22) for depression and social anxiety apps respectively. This indicates that a large segment of mental health apps for depression and anxiety are being developed by individuals who have no mental health training or connection with a relevant organization.

Only 20.48% of the apps were affiliated with an organization, university, government body, or medical center. Previous research found similarly low percentages of 35.0% (23) and 7.9% (22). While it is concerning that such a small number of apps have an institutional affiliation, it is equally concerning that the overall number of apps with published research evidence is not closer to this 20.48% figure.

In terms of cost, 74.06% of apps for depression and/or anxiety were free to download. Previous research found that 37.4% of depression apps (23), and 52.63% of social anxiety apps were free (22). This suggests that the proportion of free depression and anxiety-related apps may be increasing, which is positive for people where price-point is critical, but only beneficial if they provide valid treatment.

### Resources for Clinicians

In the absence of an adequate level of research, there are a number of places that clinicians can turn to for assistance in assessing the suitability of specific mental health apps. Firstly, efforts are being made by governments around the world to regulate mental health apps, primarily on the basis of risk of harm to the user. This is occurring in countries such as: the United States (https://www.fda.gov/medical-devices/digital-health/mobile-medical-applications), the United Kingdom (https://www.gov.uk/government/publications/health-app-assessment-criteria/criteria-for-health-app-assessment), Canada (https://www.mentalhealthcommission.ca/English/what-we-do/e-mental-health), New Zealand (https://www.health.govt.nz/our-work/digital-health/digital-health-strategic-framework), and Australia (https://www.safetyandquality.gov.au/our-work/safety-in-e-health/certification-framework-for-digital-mental-health-services/). Secondly, there are reputable websites offering information and reviews for both clinicians and consumers about mental health apps, including information on published evidence if applicable, such as: PsyberGuide (https://psyberguide.org/); the National Health Service Mental Health Apps Library (https://www.nhs.uk/apps-library/category/mental-health/); Head To Health (https://headtohealth.gov.au/); reachout.com (https://au.reachout.com/tools-and-apps); beacon (https://beacon.anu.edu.au/); and Health Navigator (https://www.healthnavigator.org.nz/apps/). Thirdly, there are at least 17 frameworks worldwide for evaluating mental health apps (33) that can be used by clinicians, including the American Psychiatric Association’s App Evaluation Model (https://www.psychiatry.org/psychiatrists/practice/mental-health-apps/app-evaluation-model).

## Limitations

This mini-review has limitations. First, there are challenges to conducting a search of the Apple App Store and Google Play, with differences in the way each delivers search results. The algorithms used in each case are unknown and remain the product of corporate intellectual property. Search results can differ on different days, and it is impossible to explain how one app can appear earlier in a search compared to another similar app. The app store searches are also limited by their minimal search options. For example, there is no filter by date, or developer, or other options as are available when searching literature using a standard database. However, analyzing the app stores remains an important exercise because this is how most people currently find mental health apps to download. While it was a deliberate methodological decision not to conduct a literature search first, and instead to search the app stores and then search the literature based on what app store descriptions revealed, it should be noted that an independent search of the literature would likely have produced more apps that met criteria as a “therapeutic treatment” for anxiety and/or depression that were not found in the app store search.

A further limitation of this study is that some of the terms used in our searches may not necessarily reflect words that consumers may use. For example, consumers may be more likely to use terms like “stress,” “depressed mood,” or “worry,” instead of terms like “dialectical behavior therapy” or “interpersonal therapy.” A third limitation of this research is that results were based on information contained in the app store description. That is, none of the apps were downloaded to ensure their store description actually matched the app’s content. Furthermore, if no research is highlighted in the app store description, this does not necessarily mean that research does not exist. This is similarly true for the issues of level of expert input and association with a relevant organization. To confirm exact numbers of apps with these elements, every app listed in each app store search would also have to be put through a literature search, which was outside the scope of this study.

## Conclusion

The proliferation of health apps, and specifically mental health apps, on the app marketplaces, such as the Apple App Store and Google Play store, has occurred without an equivalent proliferation of scientific evidence for their effectiveness. Mental health clinicians who are trained in the scientist-practitioner model of using evidence-based practice may be reluctant to use these new tools in their normal workflow, and may hesitate to recommend them to patients (34). Patients are usually ready to listen to advice from their mental health treatment providers (9), and if the evidence base for mental health apps is widened, clinicians may be more willing to recommend them (35).

The other players in a position to improve the overall situation in establishing the effectiveness of a mental health app are the app marketplaces themselves. If Apple and Google were willing to re-categorize mental health apps, and perhaps health apps generally, in a way that recognized when an app had achieved an acceptable standard of effectiveness, this would allow consumers to more easily distinguish between reliable and untested apps. From a financial perspective, Apple and Google (together with the app developers) have something to gain on this front: potentially more downloads of scientifically-tested apps because of their proven effectiveness.

Future development of mental health apps must incorporate more involvement from clinicians and institutions engaged in mental health research, training or service provision. Governments may also be able to offer regulatory oversight and certification of health apps with adequate scientific evidence for their effectiveness. Until research on the efficacy of mental health apps is broadened, and ways of searching for and recognizing effective mental health apps is improved, questions will continue to be asked about the place of these digital tools in managing mental health conditions.

## Author Contributions

JM wrote the manuscript drafts and supervised the app store review. DD and WB provided proofreading and editing, as well as supervising the overall research project. All authors read and approved the final submitted version.

## Funding

JM is in receipt of an Australian Government Research Training Program Stipend Scholarship.

## Conflict of Interest

The authors declare that the research was conducted in the absence of any commercial or financial relationships that could be construed as a potential conflict of interest.
